# Quaternary Structure Heterogeneity of Oligomeric Proteins: A SAXS and SANS Study of the Dissociation Products of *Octopus vulgaris* Hemocyanin

**DOI:** 10.1371/journal.pone.0049644

**Published:** 2012-11-15

**Authors:** Francesco Spinozzi, Paolo Mariani, Ivan Mičetić, Claudio Ferrero, Diego Pontoni, Mariano Beltramini

**Affiliations:** 1 Department of Life and Environmental Sciences, Marche Polytechnic University and CNISM, Ancona, Italy; 2 Department of Biology, University of Padova, Padova, Italy; 3 European Synchrotron Radiation Facility, Grenoble, France; University of Oulu, Finland

## Abstract

*Octopus vulgaris* hemocyanin shows a particular self-assembling pattern, characterized by a hierarchical organization of monomers. The highest molecular weight aggregate is a decamer, the stability of which in solution depends on several parameters. Different pH values, buffer compositions, H_2_O/D_2_O ratios and Hofmeister’s salts result in modifications of the aggregation state of *Octopus vulgaris* hemocyanin. The new QUAFIT method, recently applied to derive the structure of the decameric and the monomeric assembly from small-angle scattering data, is used here to model the polydisperse system that results from changing the solution conditions. A dataset of small-angle X-rays and neutron scattering curves is analysed by QUAFIT to derive structure, composition and concentration of different assemblies present in solution. According to the hierarchy of the association/dissociation processes and the possible number of different aggregation products in solution, each sample has been considered as a heterogeneous mixture composed of the entire decamer, the dissociated “loose” monomer and all the intermediate dissociation products. Scattering curves corresponding to given experimental conditions are well fitted by using a linear combination of single particle form factors. QUAFIT has proved to be a method of general validity to describe solutions of proteins that, even after purification processes, result to be intrinsically heterogeneous.

## Introduction

The quaternary structure of an oligomeric protein in solution depends on a complex network of interactions between subunits or domains and between these and the solvent. The description of such structures requires the identification of the building blocks as well as the determination of the hierarchy of the oligomer assembling [1]. This kind of study is complicated by several factors. For example, the structure of a given building block may change when it is dissociated from the interacting counterparts or it is assembled into the oligomer, due to the different solution conditions that stabilize the oligomers or dissociate the subunits (*e.g.* pH values, presence of chaotropic or cosmotropic salts). Furthermore, the interactions between subunits may be highly hierarchical and the association/dissociation patterns, which might include several intermediates, can be strongly dependent on the solution conditions [2], [3]. The thorough modelling of association/dissociation phenomena implies the use of experimental approaches that do not alter the multiple equilibria between subunits. It is however clear that the isolation of an intermediate aggregation product aiming at its structural characterization could perturb the equilibria among different aggregated forms: it is therefore necessary to rely on methods capable to describe all species existing in polydisperse systems. In this frame, small-angle scattering (SAS) measurements, obtained using X-ray (SAXS) as well as neutron (SANS) beams, are of particular importance, since all the oligomeric forms of the same macromolecule contribute to the scattering signal. As a consequence, SAS measurements do not need any fractionation of given component(s) of the mixture, which may alter the association/dissociation equilibria of interacting oligomers. As a matter of fact, there is an increasing interest in exploiting the SAXS approach for high throughput structural studies on biological macromolecules resulting in the implementation of data collection strategies and sample handling systems [4]–[7]. However, the capability of getting the whole information content from a SAS curve or a batch of SAS curves of a polydisperse system strongly depends on the availability of proper analysis tools or algorithms, in particular when the low-resolution shape of one or several components is lacking. In order to face this intriguing challenge, we have recently developed QUAFIT, a novel computer code designed for determining the quaternary structure of a protein in solution by the analysis of SAXS and SANS experiments [8]. The method is based on the idea that, according to a proper point group symmetry and/or a screw axis, the structure of the protein assembly (also called “biological unit”) is determined on one hand, through a sequence of aggregative intermediate species, by the best arrangement of an “asymmetric unit” (the monomer) and, on the other hand, by the best arrangement of the “rigid domains” that constitute the monomer.

The oxygen-transporting proteins of molluscs and arthropods, the hemocyanins, represent a paradigmatic example of giant proteins with a complex quaternary structure, which results from the aggregation of subunits through a complex pattern of interactions [2], [3], [9]. The building block of *Octopus vulgaris* hemocyanin is made up of seven asymmetric paralogous functional units (FUs) of 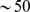 kDa (named a–g) with different sequences but similar folds. SAS techniques have largely proved to be useful for describing conformational changes of hemocyanins that occur upon binding molecular oxygen and other ligands [10]–[14]. The QUAFIT method has been successfully applied to derive the quaternary structure of *Octopus vulgaris* hemocyanin under both fully associating and fully dissociating conditions [8]. In the QUAFIT approach, the seven asymmetric FUs constituting the monomer are considered as rigid domains (RD) of a known structure, connected by flexible linkers (FL) of a known sequence.

The derived structure of the decamer is in excellent agreement with recently published models [15]–[17], based on cryo-electron microscopy (cryo-EM) low-resolution approaches, and the structure of the dissociated “loose” monomer is found to be well depicted by a distribution of many conformations. The QUAFIT calculations have been performed evaluating the relative positions and orientations of the different monomer domains, the conformation of flexible linkers among such domains and the arrangement of the monomers in the macromolecular assembly that best fit SAS data [8].

The robustness of a shape reconstruction method depends also on its applicability to polydisperse systems where more than one conformation exists in solution. This structural heterogeneity may result from the existence of different oligomers, whose abundance depends on the solution conditions, and/or on the conformational disorder, as in the case of naturally unfolded proteins or multi-domain proteins with high conformational flexibility between domains. Hemocyanins in solution represent a typical example of polydisperse system, due to their high structural heterogeneity. In particular, the subunit-subunit interactions of *Octopus vulgaris* hemocyanin are highly hierarchical and the related association/dissociation patterns showed that a number of different aggregation products could form in solution depending on buffer conditions [2], [18]–[21]. Intermediate aggregation forms, referred to as “small multimers” and “large multimers”, have been described in addition to the dimers, which represent the lower molecular weight aggregates [18]. Hofmeister’s salts and organic denaturants such as urea have been extensively used to probe the importance of hydrophobic interactions in stabilizing the quaternary structure of molluscan hemocyanins [2], [22]. Stopped flow kinetics, applied to dissociation studies of *Octopus vulgaris* hemocyanin induced by alkaline pH and removal of divalent cations, suggested octamers and dimers as kinetically detectable intermediate species in addition to the initial and final species, i.e. decamers and monomers, respectively [23]. On this basis, it appears that any perturbation of the buffer conditions produces a polydisperse distribution of different aggregation states. The same scenario also results in reassociation processes, in which the monomers represent the starting material and the buffer conditions are selected in order to stabilize the higher aggregation oligomers [22], [24]. In the light of all these findings, we exploit in this extensive work the QUAFIT capability to describe the structure of all possible oligomers of the *Octopus vulgaris* hemocyanin along distinct and complex dissociation/association pathways. This task is performed by analysing with QUAFIT a batch of SAS curves obtained in different experimental conditions in order to derive the fractions of protein forming the different intermediate species and their dependency on buffer conditions. As the main result, the heterogeneous distribution of molecular forms generated by different assemblies of building blocks with known structures is assessed and the thermodynamic stability of the dissociation patterns is obtained.

All recent structural analyses on molluscan hemocyanin subunits assembled in decamers (in the case of hemocyanins from cephalopods) or di-decamers (in the case of hemocyanins from gastropods, chitons and protobranch bivalves) have been based on cryo-EM [15]–[17]. They allowed defining the interaction pathway between functional units of two contiguous subunits and the contact areas between them. These results are implied in the present work, in which we study the aggregation state and stability of *Octopus vulgaris* hemocyanins in solution by SAS techniques against changes of buffer conditions.

## Materials and Methods

*Octopus vulgaris* hemocyanin has been purified as described in Favilla et al. [23] and stored at −20°C in 50 mM Tris/HCl, 20 mM CaCl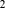, pH 7.5, in the presence of 20% (w/v) sucrose. Before SAXS/SANS measurements, an aliquot of the stock protein has been thawed and, in order to induce protein dissociation and to generate oligomers with different molecular weight, has been equilibrated by 24 h dialysis at 4°C against different buffers (50 mM Tris at pH 7.0, 50 mM phosphate at pH 7.0, 50 mM Glycine at pH 9.5), with different concentrations of the chaotropic anion SCN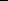, the cosmotrope F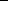, the reducing agents SO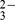 or S
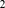
O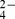, and with differently deuterated water. After dialysis, the protein solution has been centrifuged at 38,000×g at 4°C for 60 min and its concentration has been quantified spectrophotometrically at 278 nm (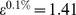 g
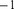
L cm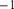). Details on the buffers used in each experimental session are given below and summarised in Tables S1, S2, S3.

### SAXS and SANS Experiments

*Octopus vulgaris* hemocyanin solutions have been analysed in four different runs of in-solution SAS experiments, three with X-rays and one with neutrons. In all cases, raw data have been radially averaged, calibrated using transmission values and detector efficiency and transformed in absolute units (cm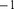). Moreover, the buffer contribution corrected for the protein volume fraction has been subtracted from the protein solution signal. The whole set of data includes ninety-one SAS curves, reporting macroscopic differential scattering cross sections, 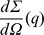, measured as a function of the modulus of the momentum transfer, 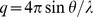 (2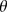 being the scattering angle and 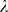 the wavelength of the incident beam). The different experimental sessions are summarised below.

#### LURE

**Figure 1:**
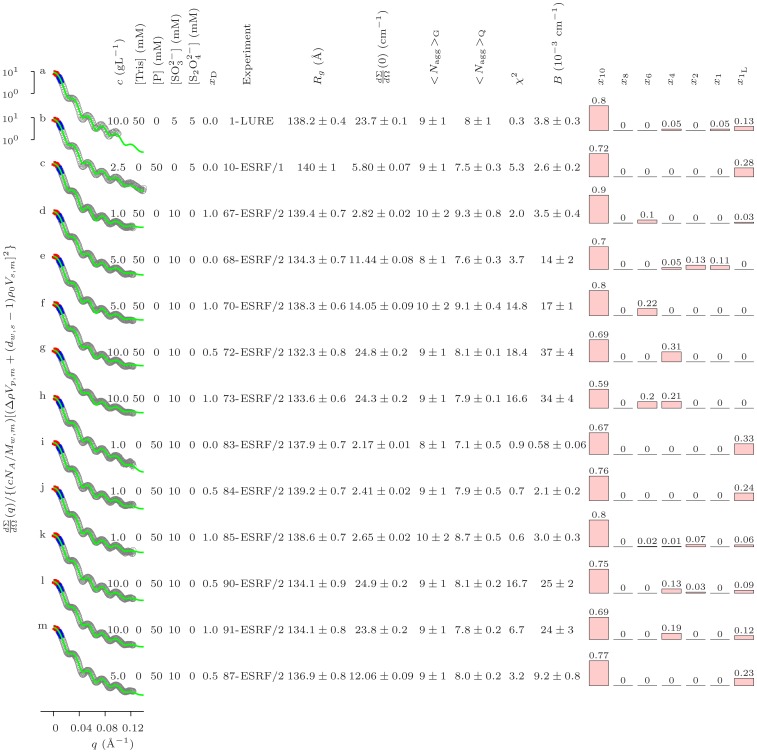
Batch of SAXS curves with the highest aggregation numbers at pH 7.0. Each experimental condition is summarised on the right-hand side of the respective SAXS pattern. The entries in column “Experiment” identify each experimental run in Tables S1, S2, S3. The solid black lines are best fitting the experimental data (grey points) normalized to the nominal concentration of the monomer and by the square of its excess scattering length. Notice that the values on the 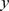-axis at 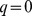 indicate aggregation numbers. Guinier approximations in the adequate 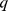-range are shown as thick blue lines, their extrapolations up to 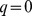 are shown as thick red lines. Histograms on the right-hand side report the protein mass fractions 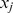 distributed in each aggregation state, as a results of the QUAFIT analysis. Uncertainties on 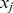 values affect the last decimal place. A zero value is assigned for 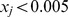.

A few SAXS curves have been recorded at beamline D24 of the LURE synchrotron (Orsay, France). In this case, *Octopus vulgaris* hemocyanin has been dissolved in a 50 mM Tris buffer (pH 7.0) to a concentration of 10.0 gL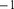, in the presence and also in the absence of 5 mM sodium dithionite and 5 mM sodium sulphite (case a defined in Fig.1 and Condition C defined in Table 1, respectively). In addition, protein samples have been measured in the same buffer, but in the presence of 10 mM sodium sulphate. All details about sample compositions are reported in Table S1 (#
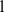
–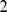).

#### ESRF/1

A second series of SAXS data has been recorded at beamline ID02 of the ESRF (Grenoble, France). In this session, the effect of chemically reducing conditions (5 mM sodium dithionite) has been investigated on *Octopus vulgaris* hemocyanin solutions at concentrations ranging from 1.0 to 10.0 gL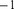, in two different 50 mM buffers with pH 7.0, Tris and phosphate (Conditions C, F and E reported in Table 1). Furthermore, the influence of increasing sodium fluoride concentration (up to 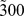 mM) has been studied on a 5.0 gL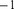 protein solution with 100 mM sodium thiocyanate in three different 50 mM buffers, Tris and phosphate with pH 7.0, and glycine with pH 9.5 (Conditions A, B and D, respectively, Table 1). Details about sample compositions are summarised in Table S1 (#
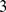
–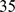).

**Table 1:** Fitting parameters of the SAXS data recorded under Conditions A–F.

	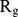	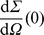	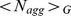	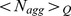	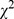	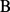	Fig.	Experiment
	(Å)	(cm 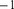 )				( 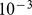 cm 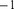 )		
Condition A (Tris/SCN)								
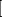 F 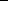 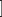 = 0 mM	60.4 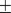 0.5	1.81 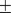 0.02	1.3 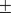 0.2	1.00 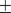 0.02	9.8	2.1 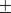 0.2	4Aa	31-ESRF/1
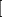 F 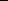 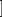 = 50 mM	64.3 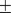 0.4	1.87 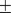 0.01	1.4 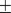 0.2	1.06 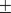 0.05	7.4	2.4 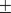 0.2	4Ab	32-ESRF/1
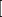 F 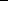 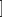 = 150 mM	72.9 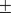 0.5	1.74 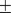 0.01	1.3 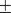 0.2	1.11 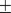 0.04	16.6	0	4Ac	33-ESRF/1
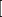 F 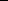 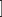 = 250 mM	85.7 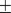 0.5	2.77 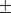 0.02	2.1 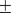 0.3	1.5 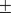 0.1	8.6	3.7 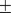 0.4	4Ad	34-ESRF/1
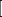 F 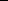 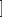 = 280 mM	85.5 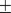 0.6	2.81 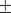 0.02	2.2 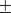 0.3	1.55 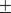 0.08	5.0	4.0 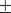 0.3	4Ae	35-ESRF/1
Condition B (Phos/SCN)								
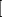 F 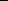 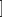 = 0 mM	125.5 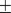 0.8	7.20 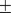 0.06	5.3 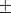 0.8	5.0 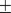 0.4	3.2	4.4 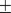 0.5	4Ba	27-ESRF/1
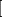 F 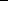 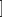 = 50 mM	125.9 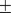 0.9	7.56 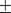 0.07	5.6 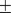 0.9	5.2 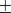 0.4	3.0	4.5 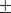 0.5	4Bb	28-ESRF/1
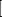 F 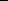 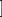 = 150 mM	122 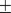 2	7.8 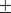 0.2	5.9 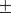 0.9	5.2 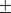 0.4	8.9	5.9 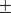 0.6	4Bc	29-ESRF/1
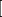 F 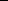 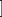 = 250 mM	123 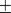 2	8.2 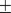 0.2	6 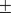 1	5.5 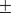 0.4	9.9	6.6 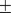 0.6	4Bd	30-ESRF/1
Condition C (Tris)								
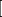 Hc 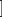 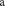 = 1.0	119.7 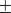 0.5	1.022 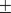 0.005	3.8 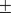 0.6	3.4 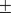 0.2	0.1	1.1 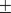 0.1	4Ca	4-ESRF/1
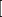 Hc 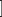 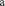 = 2.5	117.9 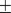 0.5	2.61 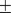 0.01	3.9 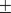 0.6	3.6 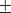 0.2	0.3	2.6 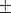 0.2	4Cb	5-ESRF/1
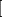 Hc 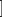 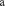 = 5.0	117.7 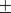 0.5	5.73 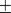 0.03	4.2 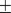 0.7	3.9 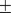 0.2	2.3	7.2 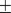 0.8	4Cc	6-ESRF/1
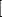 Hc 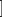 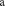 = 7.5	116.1 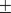 0.5	8.38 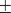 0.04	4.1 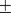 0.6	3.8 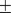 0.1	2.3	9.8 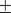 0.9	4Cd	7-ESRF/1
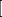 Hc 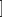 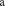 = 10.0	115.5 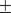 0.5	10.79 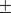 0.06	4.0 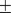 0.6	3.13 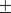 0.09	8.5	9 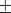 1	4Ce	8-ESRF/1
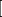 Hc 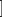 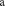 = 10.0*	106 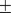 1	9.8 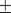 0.1	3.7 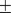 0.6	3.6 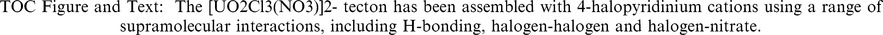 0.4	0.1	3.9 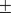 0.4	4Cf	2-LURE
Condition D (Glyc/SCN)								
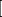 F 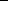 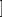 = 0 mM	78.0 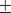 0.8	1.74 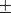 0.02	1.3 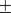 0.2	1.3 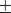 0.1	2.4	4.2 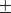 0.5	5Da	19-ESRF/1
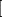 F 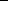 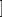 = 50 mM	78.9 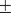 0.9	1.77 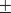 0.02	1.3 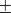 0.2	1.3 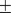 0.2	1.9	3.1 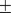 0.3	5Db	20-ESRF/1
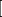 F 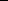 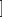 = 100 mM	75.4 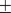 0.7	1.70 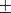 0.02	1.3 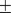 0.2	1.3 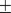 0.2	0.8	3.0 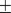 0.3	5Dc	21-ESRF/1
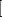 F 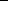 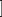 = 150 mM	71.2 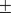 0.6	1.59 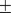 0.01	1.2 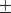 0.2	1.1 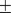 0.2	0.9	3.8 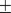 0.4	5Dd	22-ESRF/1
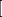 F 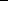 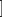 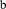 = 200 mM	71.3 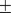 0.5	1.76 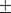 0.01	1.3 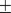 0.2	1	0.4	3.9 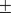 0.3	5De	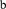 23-ESRF/1
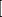 F 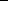 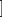 = 250 mM	67.0 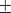 0.4	1.540 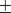 0.008	1.2 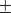 0.2	1.0 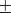 0.1	1.0	3.0 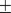 0.3	5Df	24-ESRF/1
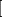 F 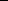 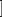 = 300 mM	99.9 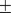 0.5	3.10 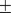 0.02	2.4 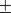 0.4	1.61 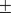 0.06	13.8	7.3 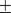 0.8	5Dg	25-ESRF/1
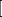 F 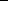 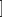 = 390 mM	125.1 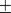 0.8	7.06 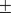 0.06	5.5 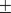 0.9	4.0 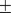 0.1	12.8	8.9 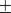 0.8	5Dh	26-ESRF/1
Condition E (Phos/S 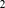 O 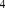 )								
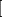 Hc 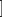 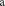 = 1.0	142 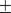 1	2.19 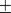 0.03	8 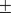 1	5.7 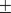 0.3	5.0	1.04 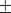 0.09	5Ea	9-ESRF/1
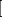 Hc 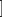 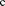 = 2.5	140 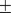 1	5.80 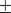 0.07	9 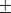 1	7.5 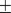 0.3	5.3	2.6 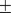 0.3	5Eb	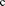 10-ESRF/1
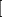 Hc 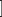 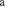 = 5.0	138.0 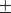 0.9	12.9 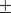 0.1	9 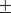 1	6.0 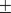 0.1	12.5	7.7 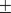 0.7	5Ec	11-ESRF/1
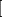 Hc 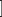 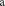 = 7.5	135.8 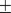 0.9	19.0 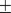 0.2	9 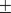 1	6.2 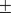 0.1	15.7	12 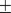 1	5Ed	12-ESRF/1
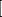 Hc 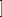 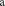 = 10.0	133 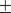 1	24.4 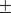 0.2	9 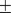 1	6.2 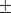 0.1	17.6	16 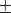 1	5Ee	13-ESRF/1
Condition F (Tris/S 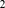 O 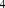 )								
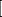 Hc 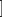 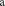 = 1.0	129.6 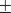 0.8	1.37 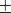 0.01	5.1 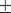 0.8	4.5 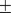 0.3	0.3	1.1 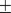 0.1	5Fa	14-ESRF/1
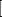 Hc 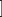 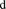 = 2.5	137.3 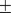 0.9	6.07 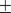 0.06	9 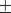 1	8 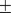 2	2.8	5.5 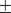 0.6	5Fb	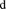 15-ESRF/1
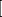 Hc 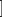 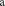 = 5.0	130.3 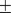 0.8	8.50 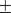 0.07	6 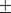 1	5.7 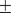 0.3	0.9	6.8 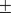 0.8	5Fc	16-ESRF/1
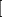 Hc 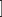 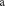 = 7.5	127.4 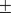 0.8	13.0 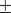 0.1	6 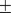 1	5.9 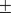 0.2	3.9	14 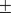 1	5Fd	17-ESRF/1
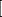 Hc 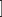 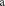 = 10.0	127 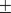 1	16.5 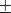 0.2	6.1 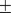 0.9	5.6 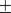 0.1	5.2	14 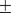 2	5Fe	18-ESRF/1

The experimental SAXS patterns are shown in various figures as indicated in the text. Condition A: hemocyanin concentration 5.0 gL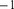, 50 mM Tris/HCl pH 7.0, 100 mM SCN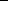; Condition B: hemocyanin concentration 5.0 gL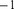, 50 mM phosphate pH 7.0, 100 mM SCN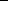; Condition C: 50 mM Tris/HCl pH 7.0; the sample marked with (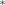) contains also 40 mM CaCl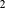; Condition D: hemocyanin concentration 5.0 gL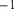, 50 mM glycine pH 9.5, EDTA 5 mM, 100 mM SCN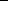; Condition E: 50 mM phosphate pH 7.0, 5 mM sodium dithionite; Condition F: 50 mM Tris/HCl pH 7.0, 5 mM sodium dithionite. The entries in column “Experiment” identify the experimental runs in Tables S1, S2, S3; 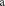 [Hc]: hemocyanin concentration in gL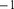; 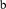 as from Fig. 2a of Ref. [8]; 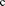 as from Fig. 1 curve b; 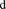 as from Fig. 2c of Ref. [8].

#### ILL

SANS experiments have been carried out, too. Measurements have been performed at the D11 instrument (ILL, Grenoble, France) with fixed protein concentration (10.0 gL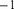) in two 50 mM buffers with pH 7.0 (Tris and phosphate), with and without chemically reducing conditions (obtained with 10 mM sodium sulphite) and by using five solution deuteration grades from 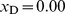 to 1.00, 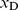 being the heavy-water-to-heavy-and-light-water molar ratio, 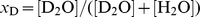. Details about sample compositions are reported in Table S2 (#
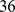
–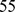) in the Supporting Information. These data refer to Conditions from G to J reported in Table S4.

#### ESRF/2

In order to further investigate the effects of solvent deuteration, a second series of SAXS experiments has been performed at the ID02 beamline (ESRF, Grenoble, France). In this case, protein concentrations ranged from 1.0 to 10.0 gL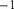. As before, two 50 mM buffers at pH 7.0 (Tris and phosphate) have been used, with and without 10 mM sodium sulphite and at 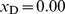, 0.50 and 1.00. All details on sample compositions are reported in Table S3 (#
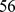
–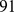). These data refer to Conditions from K to V in Table S5.

### Methods

Data have been first evaluated according to the Guinier law, 
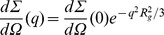
, which allows to estimate the particle gyration radius, 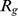, and the forward scattering cross sections, 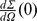. Consequently, for each experimental condition, the average aggregation number has been determined, according to 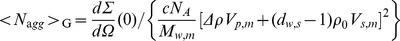 [8]. The first factor, 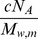, is the nominal hemocyanin concentration (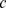 being the w/v protein concentration, 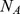 the Avogadro number and 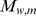 the molecular weight of the monomer), whereas the other factor, 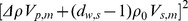, is the squared excess scattering length of the monomer, where 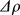 is the difference between the scattering length density of the protein (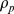) and of the solvent (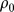), 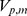 and 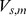 are the estimated values of the core monomer volume and its first solvation shell, respectively, and 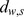 is the relative mass density of hydration water, here fixed to 1.10 [25].

The scattering curves, normalized by the two factors introduced above, have been then analysed by Singular Value Decomposition (SVD) [26]. This statistical method, mainly used in spectroscopy, allows the “unbiased” determination of the minimum number 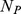 of linearly independent functions, whose linear combinations can reproduce all the curves of a dataset [27], [28]. Here, 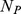 can be considered as the number of pure species simultaneously present in the analysed mixtures.

**Figure 2:**
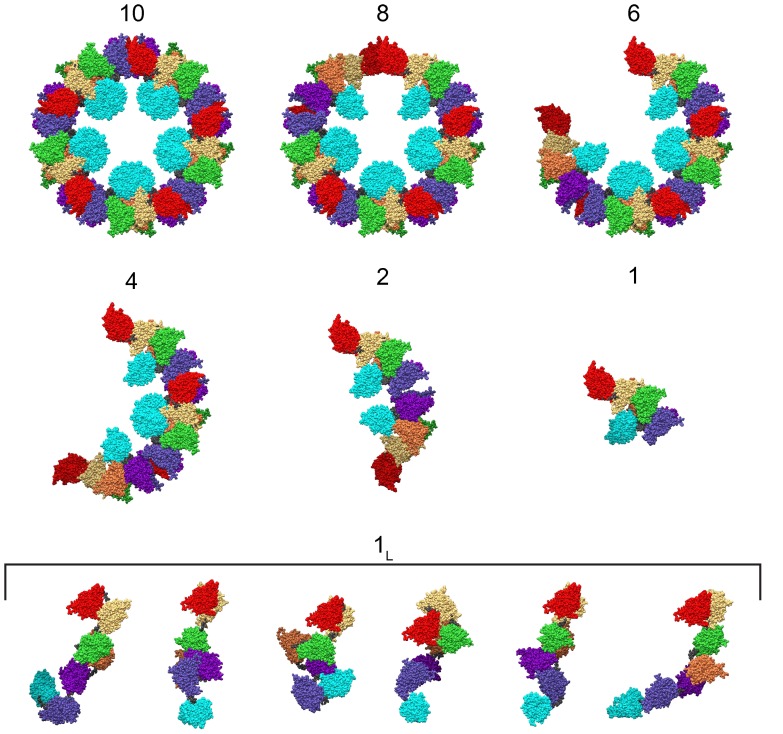
Molecular views of *Octopus vulgaris* hemocyanin in the different aggregation states exploited by QUAFIT to analyse all SAS curves: the “compact” monomer (1) (see Fig. S12 of Ref. [**8**]) and its hierarchical association products, the dimer (2), the tetramer (4), the hexamer (6), the octamer (8) and the associated full 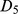
 **-dimer (10) (see Fig. 3 of Ref.** [**8**] **); the dissociated “loose” monomer (** 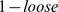 **) represented by six conformations (see Fig. S11 of Ref.** [**8**] **).** The seven rigid domains (a–g) are colour-coded according to Ref. [15] and the six flexible linkers are shown in dark grey.

The QUAFIT method has been finally used to derive structure, composition and concentration of different assemblies present in solution. According to the hierarchy of the association/dissociation processes and the possible number of different aggregation products, each sample has been considered as a heterogeneous mixture made up of the entire decamer, the dissociated “loose” monomer and all the intermediate decamer dissociation products. The corresponding SAS curves have been then fitted by using a linear combination of single particle form factors, 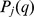, as expressed by Eq. 1 [8]:
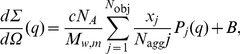
(1)where 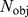 is the number of all the possible particles in solution (including monomers, intermediates as well as entire oligomers), 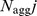 is the aggregation number of the 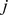-particle and 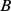 is a flat background term, which can take into account incoherent scattering processes as well as improper buffer subtractions [29]. The form factor of the entire decamer and the one of the dissociated “loose” monomer are taken from the results of Ref. [8]: the decamer is built by combining ten “compact” monomers in the symmetry positions of the 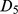 point group, whereas the dissociated “loose” monomer is described by a mixture of six “pearl necklace” conformations. Conversely, in the present work, the form factors of the intermediate products arising from the dissociation of the entire decamer are calculated with QUAFIT on the basis of the same “compact” monomer, but considering the point group 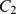 for the dimer and 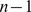 roto-translational steps around a screw axis perpendicular to the symmetry axis of 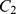 (by fixing 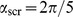 and 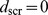, see the paragraph “Oligomerization intermediates” of Ref. [8]), with 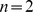, 3 and 4 for the tetramer, the hexamer and the octamer, respectively. Indeed, the 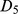 decamer symmetry can be decomposed into a combination of a 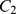 and a 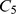 group with the two mutually perpendicular axes of symmetry. A molecular view of all species supposed to be present in the mixtures is reported in Fig. 2.

According to Eq. 1, the key curve fitting parameters are the set of 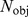 fractions, 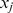 (with the constraint 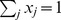), which express the amount of hemocyanin monomers that build up the 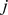-particle, together with the flat background, 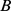, of little interest. According to the expected dissociated and partially dissociated oligomeric forms (Fig. 2), label 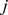 will be 10, 8, 6, 4, 2, 1 and 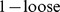, for decamer, octamer, hexamer, tetramer, dimer, “compact” monomer and “loose” monomer, respectively. Note that the values of 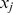 optimized by the QUAFIT analysis can be utilised to calculate the average aggregation number, according to 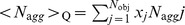 (see Eq. 19 in Ref. [8]), which can be compared with the corresponding Guinier aggregation number as a first reliability check of the method. It is also worth noticing that the uncertainty on the 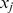 values is strongly reduced by the availability of SAS data measured on an absolute scale. In addition, the particle fractions 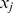 allow the evaluation of the chemical potentials for the hemocyanin monomer in each state according to
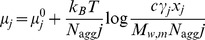
(2)where 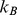 is the Boltzmann constant, 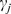 the activity coefficient of the 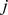-particle and 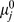 the chemical potential of one monomer inside the 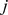-particle in the standard state, defined as the solution of 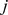 at concentration 1 M in which all interactions among all the other particles are virtually suppressed. The equilibrium condition is established when the chemical potentials 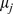 of a monomer in any 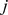 species have the same value. For each sample condition investigated by SAS, the thermodynamic stability 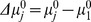 of each state 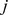 with respect to the dissociated “compact” monomer state (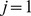) can hence be described using the relation:



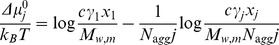
(3)In this context, it should be noticed that, at the protein concentrations used in the SAS experiments, the values of 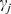, which we have calculated using the scaled particle theory for hard sphere mixtures [30], are very close to unity, indicating a substantially ideal behaviour of the system.

## Results and Discussion

**Figure 3:**
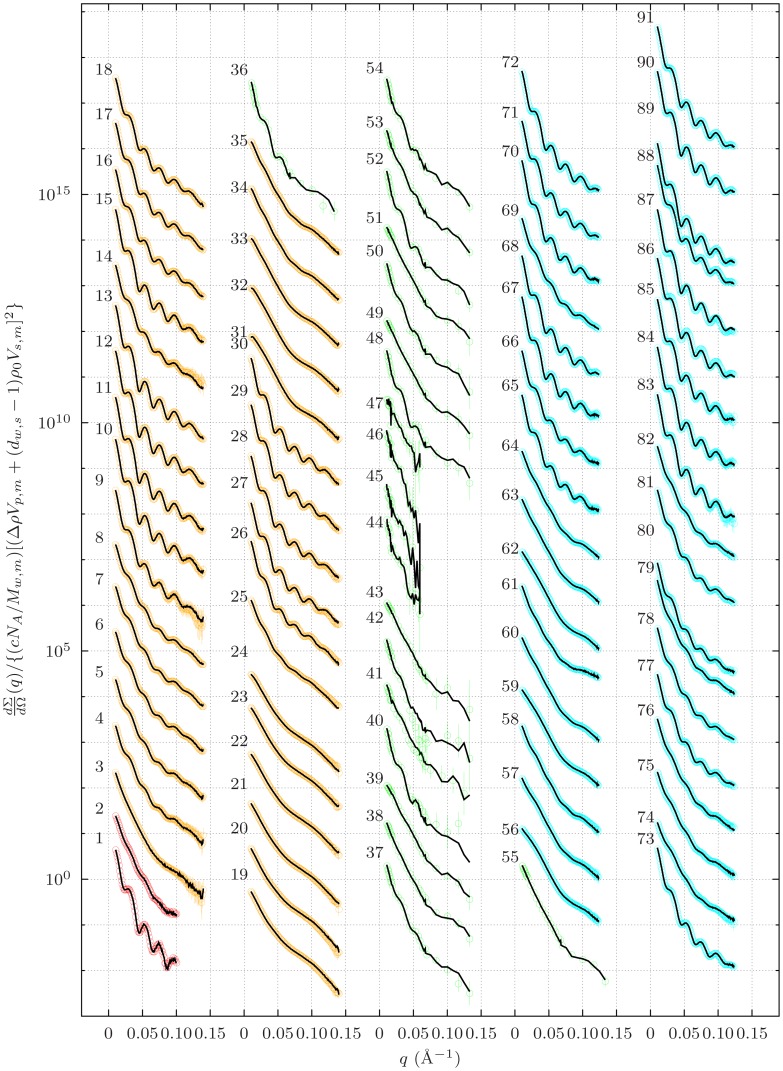
SAS curves recorded from *Octopus vulgaris* hemocyanin samples in four experimental sessions: synchrotron radiation X-rays at LURE (red points 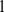
 **–** 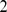 **) and at the ESRF in two separate sessions (ESRF/1, orange points** 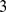 **–** 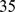 **and ESRF/2, cyan points** 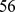 **–** 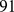 **); neutrons at the ILL (green points** 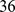 **–** 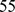 **).** The data are macroscopic differential scattering cross sections, normalized to the nominal concentration of the hemocyanin monomer and to the square of its excess scattering length, upscaled by successive multiples of 10, and reported versus the momentum transfer 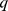. Notice that the values on the 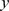-axis at 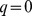 represent aggregation numbers. The solid black lines are linear combinations of seven basis functions found by SVD for each of the four sets of curves.

The present work is based on a dataset of ninety-one SAXS and SANS patterns, as summarised in Tables S1, S2, S3. As evident from Fig. 3, where all the measured SAXS and SANS curves are reported, the composition of the solution (protein concentration, pH, buffer type, presence of chaotropic or cosmotropic anions, presence of reducing agents and deuteration grade) is reflected in characteristic scattering patterns, clearly indicating that the aggregation state of the *Octopus vulgaris* hemocyanin is affected by the solution conditions. Looking to the curves in the lowest 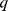-range, neither interference peaks nor trends with positive concavity can be seen, indicating the absence of any attractive or repulsive interparticle effect.

### Guinier Analysis

Guinier plots (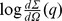 *vs.* 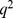) of the SAS patterns show a linear trend at low 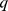, confirming that interparticle structure factors are essentially negligible for any of the investigated conditions. The datasets include thirteen cases (highlighted in red colour in Tables S1, S2, S3) characterized by strong modulations with several minima and maxima along the 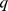 axis and consistent with a dominant presence of decameric oligomers. A curve representative of these patterns has been used to reconstruct the quaternary structure of decameric protein using QUAFIT in a previous work, (see Ref. [8], Fig. 2c). Such patterns have been observed in Tris/HCl or phosphate buffers with pH 7.0 in the presence of reducing agents, such as dithionite, sulphite or both (Fig. 1, curves b, c–m, and a, respectively). It is worth noting that the SAXS profiles appear unrelated to the investigated protein concentrations (from 1.0 to 10.0 gL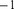) and to the presence of heavy water in solution, cfr. *e.g.* the 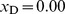 curves shown in Fig. 1 (curves a, b, d and h) with the 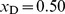 and 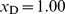 curves (curves f, i, k and m and c, e, g, j and l, respectively). Guinier analysis provided the 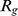 (radius of gyration) values reported in Fig. 1: it can be seen that 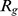 ranges from 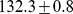 to 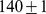, while the derived values of 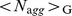 are in the 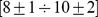 range, clearly indicating the presence to a large extent of decamers to a large extent, together with a few dissociation products. In contrast, ten cases (highlighted in blue in Tables S1, S2, S3) result in smooth SAXS patterns. The structure of *Octopus vulgaris* hemocyanin under these conditions, modelled by QUAFIT in [8], corresponds to that of the protein in the monomeric form. The monomer can be depicted as indicated in Fig. 2, structures 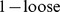 [8]. The structures of both decameric and monomeric hemocyanin represent reference points for the analysis of the oligomeric heterogeneity presented in this paper, focused on the evaluation of the average aggregation number from both the Guinier analysis (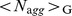) and QUAFIT (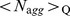).

**Figure 4:**
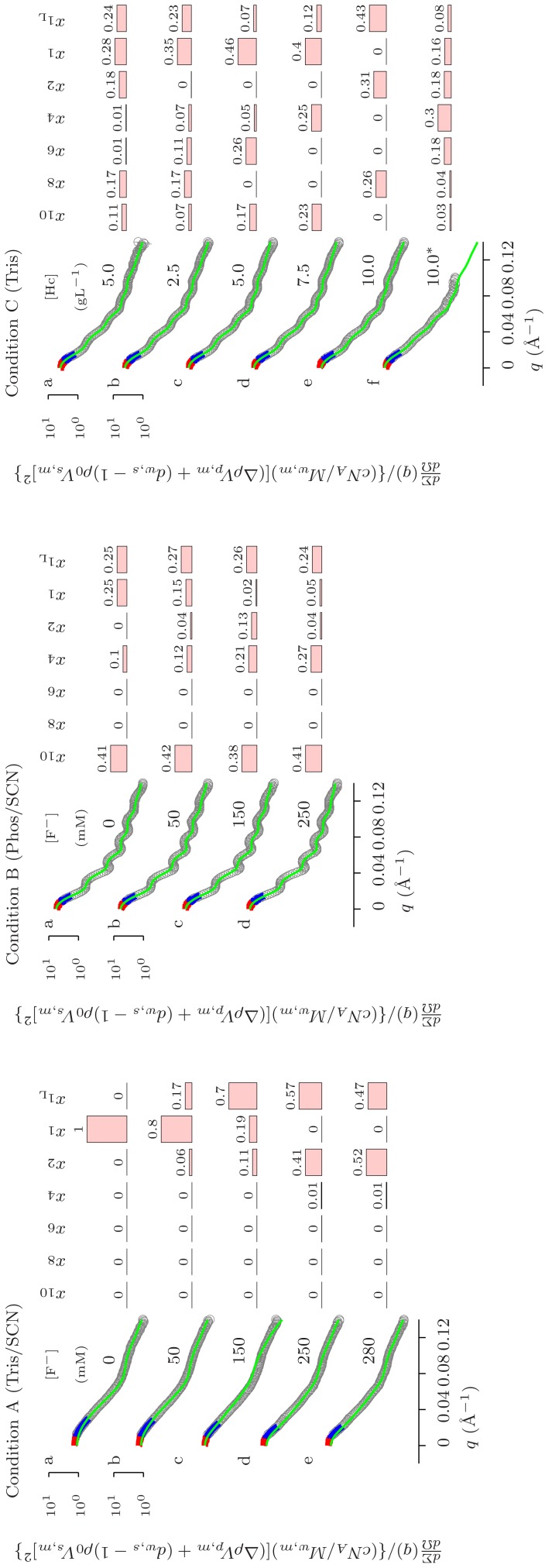
SAXS curves referring to *Octopus vulgaris* hemocyanin in Conditions A, B and C. Condition A: hemocyanin 5.0 gL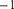 in 50 mM Tris/HCl pH 7.0 upon increasing F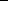 concentration in the presence of 100 mM SCN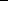. Condition B: hemocyanin 5.0 gL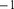 in 50 mM phosphate pH 7.0 upon increasing F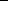 concentration in the presence of 100 mM SCN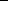. Condition C: hemocyanin in 50 mM Tris/HCl pH 7.0 upon protein concentration increasing as indicated. The sample marked with (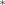) contains also 40 mM CaCl_2_. Results of the Guinier analysis for these conditions are reported in Table 1. See the caption of Fig. 1for more details.

**Figure 5:**
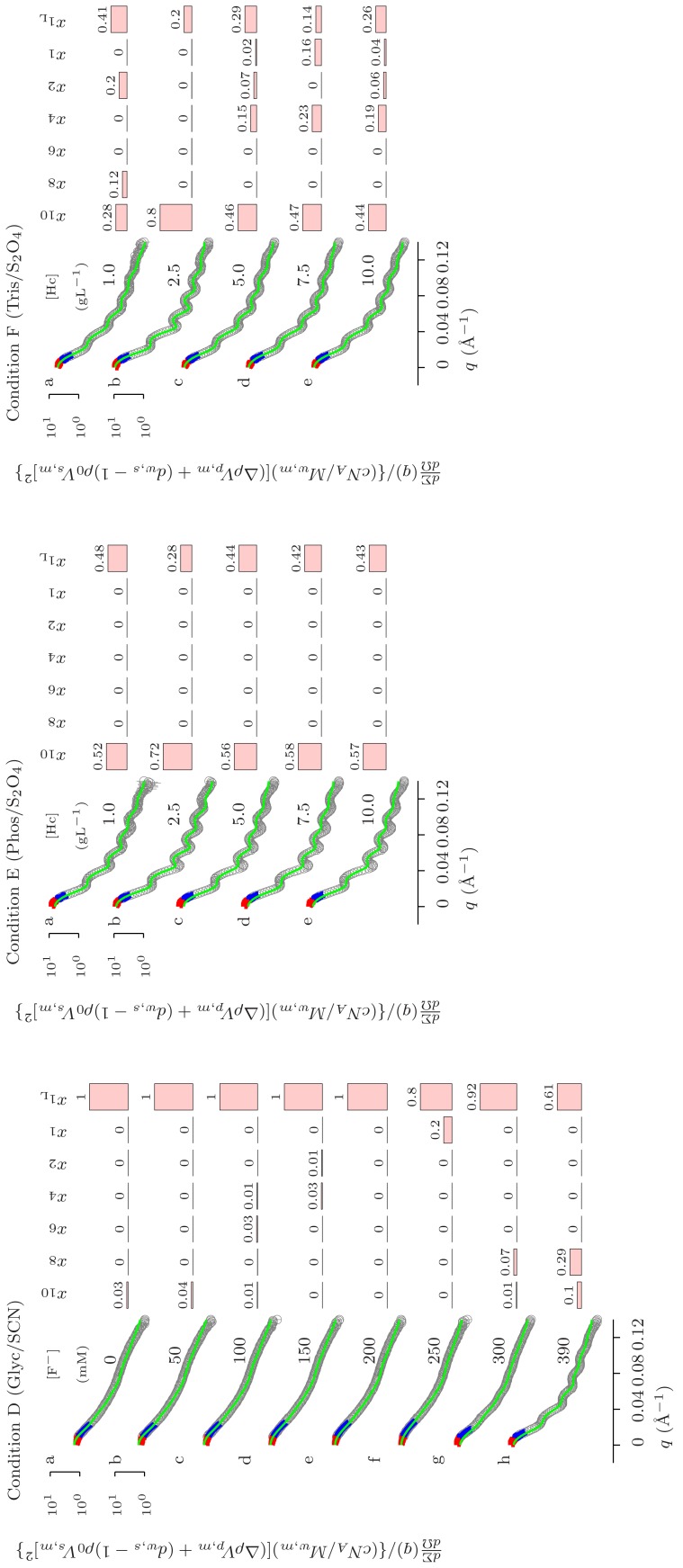
SAXS curves referring to *Octopus vulgaris* hemocyanin in Conditions D, E and F. Condition D: hemocyanin 5.0 gL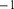 in 50 mM glycine pH 9.5, EDTA 5 mM, 100 mM SCN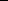 upon F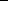 concentration increasing as indicated. Condition E: hemocyanin in 50 mM phosphate pH 7.0, 5 mM sodium dithionite upon protein concentration increasing as indicated. Condition F: hemocyanin in 50 mM Tris/HCl pH 7.0, 5 mM sodium dithionite upon protein concentration increasing as indicated. Results of the Guinier analysis for these conditions are reported in Table 1. See the caption of Fig. 1 for more details.

For example, smooth patterns characterized by the absence of the distinctive modulations already observed for intact decamers, are detected for *Octopus vulgaris* hemocyanin dissolved in a Tris/HCl buffer pH 7.0, 100 mM SCN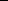 and in the presence of increasing concentrations of F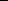 up to 280 mM (Fig. 4, Condition A). The formation of dissociation products is also confirmed by the corresponding 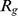 values, which are smaller than those measured in the presence of intact decamers. As summarised in Table 1 (Condition A), 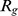 changes from 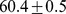 Å (absence of F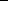) to 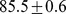 Å (280 mM F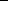), suggesting that the distribution of dissociated forms depends on the buffer composition. In particular, 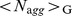 is found to be around 1 (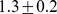 in Tris/HCl buffer pH 7.0, 100 mM SCN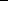, and slightly increasing up to 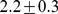 when the F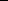 concentration increases), then pointing out the presence of mainly dimeric aggregates. Similar SAS patterns, in which the more or less evident modulations suggest different distributions of dissociated and partially dissociated oligomers, are also shown in Fig. 4, Conditions B and C, and in Fig. 5. The corresponding 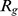 and 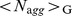 values (see Table 1) corroborate this behaviour.

### SVD

The SVD method has been applied to each of the four sets of curves collected during the different SAS experiments (see the paragraph “SAXS and SANS experiments”). As a result, the number 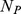 of pure species involved in different mixtures has been found to be seven. In Fig. 3, the solid black lines are the curves re-calculated as linear combinations of the 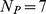 basis functions found by SVD: a total agreement with the experimental points can be easily appreciated. The SVD analysis is fully consistent with the results of the kinetic study of the *Octopus dofleini* assembly reported in Ref. [24], which clearly shows that the aggregation process is at first due to the formation of dimers, which are considered the nucleation products, followed by the fast generation of small and large multimers, up to the decamer. The dimers are constituted by the association of two “compact” monomers and have been reported as “men-in-the-boat” structures [15], [31]. In this context, it is also worthwhile noticing that most SAS curves in the dataset show more or less marked oscillations, which fall at similar 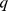 values. Such a result strongly indicates that the periodicity of the intra-particle structure is essentially maintained also when the aggregation number is lower than 10. As a consequence, we suggest that the seven species present in solution, as detected by the SVD analysis, could be represented by the decamer, by its five hierarchical dissociation intermediates [23] (octamer, hexamer, tetramer, dimer and “compact” monomer) and by the dissociated “loose” monomer [8], as shown in Fig. 2(note that the dissociated “loose” monomer is described by the combination of six representative conformations [8]). To confirm this hypothesis, we have then analysed each curve applying the QUAFIT method, as detailed in the paragraph “Methods”. The final results of the analysis are discussed hereafter.

### QUAFIT Analysis

The whole set of ninety-one SAS patterns has been analysed by the QUAFIT method to confirm the hypothesis put forward in “SVD”. All best fitting curves are reported in Fig. S1. The results, classified according to different solution Conditions, are described in detail in the following paragraphs.

#### Mostly aggregative Conditions

The SAXS data with the strongest modulations are shown in Fig. 1. The good quality of the best fit results can be directly appraised considering the excellent superposition of any best fit curve on the experimental points. In particular, the modulations appear to be very well reproduced. Moreover, the average aggregation numbers calculated via QUAFIT and separately determined by the Guinier approximation occur to be very similar. The main fit parameters are reported in Fig. 1 in form of histograms showing the fraction 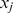 of *Octopus vulgaris* hemocyanin monomers distributed in each aggregation state. The presence of decamers is clearly confirmed, even if a small fraction of “loose” monomers characterizes the sample prepared in the phosphate buffer, while the presence of D
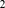
O induces the formation of large, partially dissociated oligomeric states. The latter finding is not obvious, at least according to the known protein hydration changes in D
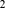
O compared to H
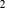
O and in the light of the complex effects on the structural stability of proteins when they are dissolved in D
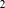
O and H
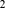
O. The hydrophobic effect is enhanced in D
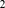
O, due to the stronger association between water molecules: indeed 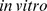 studies have provided various results about the stabilizing influence of D
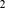
O on proteins, but in a few cases stability remained unchanged or even decreased [32], [33].

#### Conditions A and B: effect of fluoride in Tris/HCl or phosphate buffer and thiocyanate

**Figure 6:**
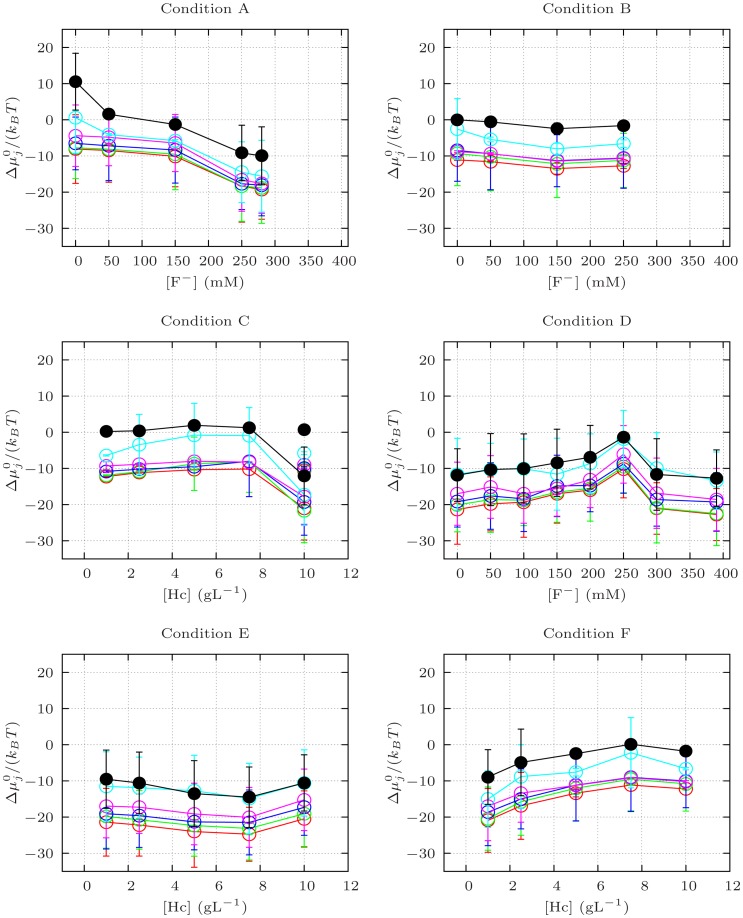
Chemical potential differences between the monomer in the 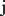
 **state and the monomer in the dissociated “compact” state (** 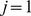 **) calculated from the fractions** 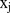 **for Conditions A to F as defined in** **Table 1** **.** Filled black circles: decamer (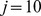); open red circles: octamer (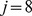); open green circles: hexamer (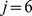); open blue circles: tetramer (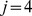); open magenta circles: dimer (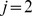); open cyan circles: dissociated “loose” monomer (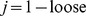).

As reported above, SAS data for hemocyanin samples prepared in Tris/HCl pH 7.0 and in the presence of SCN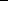 (Condition A of both Table 1 and Fig. 4) suggest the complete dissociation of the oligomers. Moreover, the addition of increasing concentrations of F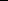, up to 280 mM, does not modify the SAS pattern features. The QUAFIT analysis clearly shows that the protein solution becomes polydisperse with increasing F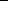 concentration. At large concentrations, dimeric aggregates are produced (namely, aggregated species formed by the interaction of two subunits, each containing seven functional units). This fact is confirmed by a decreasing trend in chemical potential differences, 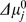, versus the F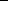 concentration, as reported in Fig. 6, Condition A: all species exhibit a lower free energy than the one of the dissociated “compact” monomer. Although it is not easy to understand the complex interplay of equilibria, the overall result is an increased concentration of the dimer and of the dissociated “loose” monomer. The middle panel of Fig. 4 depicts the QUAFIT analysis of SAXS results from *Octopus vulgaris* hemocyanin samples prepared according to Condition B (phosphate buffer pH 7.0, thiocyanate and increasing F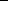 concentration, see Table 1). The histogram clearly indicates that “loose” and “compact” monomers are still present, but decamers as well as tetramers and dimers form mainly at the expenses of “compact” monomers. Aggregation appears to be more favoured by a phosphate buffer rather than a Tris/HCl buffer.

#### Condition C: effect of concentration in Tris/HCl buffer

Effects due the removal of reducing conditions can be appreciated in Fig. 4, right panel, where data refer to hemocyanin dissolved in Tris/HCl buffer (pH 7.0) at different protein concentrations (from 1.0 to 10.0 gL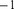). In these conditions, the modulations typical of the decamer are less pronounced, but still visible. Guinier fitting parameters are reported in Table 1 (Condition C): the gyration radii (extending from 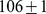 to 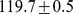 Å) do not depend on protein concentration (at least in the range explored in the experiment) and are intermediate between those of decameric hemocyanin and dissociated material. The 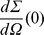 values increase from 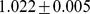 cm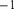 (1.0 gL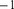) up to 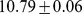 (10.0 gL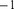) while there is not a clear correlation between protein concentration and 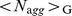. These data are difficult to rationalize but all of them suggest that high polydispersity is present and is probably related in a complex way to the protein concentration. QUAFIT results point out the formation of decamers, however they confirm the wide polydispersity of the partially dissociated forms, with a surprising slight preference for larger distribution among the possible aggregate species at low protein concentrations. Comparing these data with the ones obtained under the same conditions in the presence of chaotropic anion thiocyanate (Fig. 4, Condition A), it can however be concluded that the hemocyanin assembly is strongly hindered by chaotropic agents. Wide polydispersity is also detected in the presence of 40 mM Ca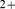, a cation known to stabilize oligomeric species in several hemocyanins (Fig. 4, Condition C, curve f). The result is unexpected, even if the histogram distribution suggests the formation of mainly intermediate species. A further confirmation of the difficulty in rationalizing the results can be inferred by observing the variations of 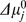, shown in Fig. 6, Condition C. As in Condition C, there is only a variation of the protein concentration, thus we expected to get constant values for the chemical potentials. On the contrary, we obtained rather oscillating values, with more pronounced changes for the highest hemocyanin concentrations that cannot be explained as yet.

#### Condition D: effect of fluoride in glycine buffer and thiocyanate

The combined effect of SCN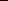 and F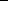 on the aggregation state of hemocyanin (similarly to the case described in Fig. 4, Conditions A and B) has been also probed in glycine buffer with pH 9.5 (100 mM thiocyanate in 50 mM glycine buffer pH 9.5 containing 5 mM EDTA and increasing F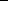 concentration up to 390 mM, see Table 1, Condition D). The SAXS patterns are displayed in Fig. 5, Conditions D. Guinier data (see Table 1) and QUAFIT fitting results are a clear signature that the experimental conditions are strongly dissociating and the “loose” monomer is the predominant species up to 200 mM F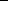. Indeed, the distribution histogram shows that larger oligomeric species (and in particular the entire decamer) becomes populated to some extent at larger investigated F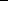 concentrations. This result confirms the “structure maker” nature of the fluoride anion in the Hofmeister series.

#### Conditions E and F: effect of concentration in Tris/HCl or phosphate buffer and dithionite

The presence of different aggregation forms is often stated in hemocyanin preparation. Actually, the purification of decamers implies the use of chromatographic techniques to remove low-molecular weight dissociated material. In order to describe the composition of the different species, we have also measured by SAXS *Octopus vulgaris* hemocyanin samples obtained without further purification of different hemocyanin oligomers. The SAXS results recorded at different protein concentrations (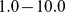 gL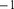) in Tris/HCl or phosphate buffers, pH 7.0 and in the presence of dithionite as reducing agent can be seen in Fig. 5 (the middle panel refers to the phosphate buffer, the right panel to the Tris/HCl buffer, Conditions E and F, respectively). In the phosphate buffer the SAXS patterns are quite similar, and, accordingly, the related 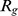 values are almost identical (see Table 1). However, an increasing trend in 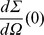 is stated as a function of protein concentration, in contrast to 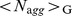 and 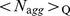, which remain constant. It should be noted that particle aggregation numbers are average values calculated over a distribution of oligomers. An in-depth analysis of polydispersion can elucidate the apparent discrepancies. The same consideration applies also to samples in the Tris/HCl buffer. In this case, SAXS patterns display less pronounced modulations than in the former case, and lower values for all parameters are obtained. QUAFIT allows explaining these data in terms of different distributions of oligomers. Indeed, in phosphate only decamers and “loose” monomers are revealed (and changes in 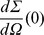 are then related to changes of the protein concentration), while in the Tris/HCl buffer the distribution of dissociated and partially dissociated species clearly depends on hemocyanin concentration. Trends of 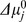 with emocyanin concentration (shown in Fig. 6, Conditions E and F) confirm the different behaviour in the two buffers: in phosphate, 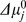 values are systematically lower than those in Tris, suggesting that in phosphate the ensemble of equilibria is shifted toward higher association products.

#### Discussion

From the whole host of results, it can be concluded that the presence of decamers (and high molecular weight aggregates) is promoted by phosphate used as a buffer with pH 7.0 (irrespective of the addition of different agents, such as the chaotropic anion SCN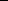, the reducing S
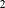
O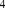 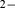 or the cosmotrope F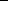). The structure-breaking effect of SCN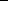 is therefore largely balanced by the cosmotropic nature of phosphate. As opposed to that, the presence of thiocyanate both in Tris/HCl pH 7.0 or in glycine pH 9.5 buffers induces a strong dissociating environment, and SAXS data show that *Octopus vulgaris* hemocyanin mainly occurs as “loose” and/or “compact” monomers. Interestingly, the addition of fluoride ions triggers the formation of associated states. Strong aggregation causing the formation of decamers is also detected in Tris/HCl after addition of dithionite.

However, it is not easy to identify the factors that control the distribution of hemocyanin molecules in various dissociated or partially dissociated states. The unusual trends of chemical potentials, even when the only varying parameter is the protein sample concentration, suggest that some other variables, which probably we do not keep under control and which influence the different equilibria in solution, have been changing in the various samples under investigation.

As an example of this complexity, the effect of isotope substitution previously discussed can be taken as a case study. Actually, the QUAFIT analysis of SANS and SAXS curves obtained from the same hemocyanin sample dissolved in mixtures of D
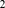
O and H
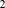
O at different compositions (Conditions from G to J and from K to V in Tables S2 and S3), testifies different oligomer distributions. The stabilization of the decamer structure stimulated by the reducing agent (sulphite, in this case) is clear, while the effect of D
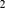
O in changing the equilibria of the system is rather irregular, as indicated by the trends of chemical potential differences reported in Fig. S2. In particular, aggregation effects in phosphate and in Tris/HCl buffers appear to be different. Under the former condition, where decamers are already present in H
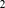
O, the addition of D
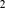
O induces the formation of large aggregates, even if the effect remains only slightly appreciable in the presence of sulphite. By contrast, the amount of decamers or large partially dissociated species does not change in Tris/HCl buffers by increasing 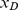, neither in the absence nor in the presence of sulphite. The predicted stabilizing effect of D
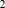
O due to an enhanced hydrophobic effect, if any, is therefore very small.

### Conclusion

*Octopus vulgaris* hemocyanin features a very particular self-assembling pattern, characterized by a hierarchical organization of “compact” monomers. The final product is a decamer, the stability of which in solution depends on several parameters, like the presence of reducing agents, pH values, subtle effects of various ions belonging to the so-called Hofmeister series dissolved in the solution, and the H
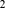
O/D
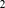
O solvent isotope variation.

This study reports on an in-solution SAXS and SANS data analysis of hemocyanin polydispersity induced by very different experimental conditions. The monitoring and comparison through the QUAFIT method of the distribution of the dissociated and partially dissociated species in all the investigated conditions is straightforward. In particular, the two extreme solution states are represented on end by “one” (all molecules are in the monomeric state) and on the other end by “ten” (all molecules are in the decameric state). In the present analysis the monomeric form can exist in two different states, *e.g.* the “loose” and the “compact” form [3]. The former is equivalent to the “necklace” or “string of beads” structure revealed by electron microscopy analysis [3] which is here accounted for by the six representative configurations described in Ref. [8]. These configurations coincide with the final structural packing the monomer exhibits when it is included in the decameric molecule [15]. The QUAFIT analysis is hence based not only on aggregation/dissociation equilibria but also on the existence of different states of the monomeric form. Intermediate aggregation states are poorly populated, however it is worth noting that the structure of the dimer formed by aggregation of two “compact” monomers is consistent with the typical “men-in-the-boat structure” (Fig 2, state 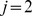) found by electron microscopy for the same species when viewed from the top [15], [31]. While all possible oligomeric forms are allowed for by QUAFIT, the low relative abundance of some intermediate oligomers strongly indicates that the association/dissociation processes are highly cooperative, monomers, dimers and decamers being the most stable species. This result is in agreement with previous kinetic evidence on the dissociation of decamers [23] and the assembly of subunits [24].

Finally, some comments are due on the results obtained for samples containing reducing agents. Actually, sulphite or dithionite can react also with molecular oxygen in the bulk, thus the concentration of oxygen may vary as a function of three factors: the presence of an excess of reducing agent, the equilibration of the bulk solution with air, and of the incubation time. Hemocyanin is an oxygen binding protein, which exists both in the oxygenated and deoxygenated state, depending on the oxygen concentration in the bulk. As a consequence, the equilibrium between oxygenated and deoxygenated hemocyanin is likely to change owing to the presence of these compounds. No control of oxygen concentration and oxygenation state of the protein has been targeted during the SAS measurements. The issue, whether within a polydisperse protein preparation the oligomeric state of *Octopus vulgaris* hemocyanin is preferentially correlated with the oxygenated or rather with the deoxygenated form (under reducing conditions) requires further investigations. SAXS-based evidence indicates that the conformation of oxy-hemocyanin differs from that of the deoxy-form in the case of both molluscan and arthropod hemocyanins [11] and SAXS-based three-dimensional reconstruction of the immunogen KLH1 reveals different oxygen-dependent conformations [13]. These differences do not alter the oligomeric state of the protein, yet they are likely to modify stability versus physical-chemical conditions of the bulk solution [34], as found also in a comparison with copper-free hemocyanin [35], [36].

## Supporting Information

Figure S1
**QUAFIT analysis of the SAS curves recorded from** ***Octopus vulgaris*** **hemocyanin samples in four experimental rounds: synchrotron radiation X-rays at LURE (red points** 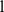 **–** 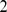 **) and at the ESRF in two separate sessions (ESRF/1, orange points** 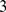 **–** 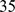 **and ESRF/2, cyan points** 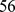 **–** 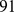 **; neutrons at the ILL (green points** 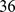 **–** 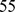 **).** The data are macroscopic differential scattering cross sections, normalized to the nominal concentration of the hemocyanin monomer and to the square of its excess scattering length (see Fig. 3) reported versus the momentum transfer 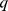. Notice that the values on the 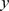-axis at 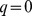 correspond to aggregation numbers. The solid black lines are the best fits obtained with the QUAFIT method. The fitting parameters are reported in Fig. 1, 2, 3, 4, 5 and in Tables 1, S4–S5.(EPS)

Figure S2
**Chemical potential differences between the monomer in the** 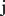 **-state and the monomer in the dissociated “compact” state (** 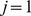 **) calculated from the fractions** 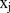 **for Conditions G to J (SANS at the ILL, see Table S4) and compared with SAXS results in the same conditions (Conditions V, P, S and M, ESRF/2, see Table S5).** Filled black triangles and circles: decamer (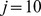), SANS and SAXS measurements, respectively; open red triangles and circles: octamer (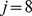); open green triangles and circles: hexamer (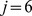); open blue triangles and circles: tetramer (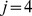); open magenta circles: dimer (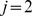); open cyan triangles and circles: dissociated “loose” monomer (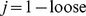).(EPS)

Table S1 **Detailed list of all chemical-physical conditions of the samples measured during the LURE and ESRF/1 campaigns.** Red and blue colours highlight experimental conditions with the strongest and the lowest modulations, respectively [8].
(PDF)

Table S2 **Detailed list of all chemical-physical conditions of the samples measured at the ILL.**
(PDF)

Table S3 **Detailed list of all chemical-physical conditions of the samples measured during the ESRF/2 campaign.** Red colours highlight experimental conditions with the strongest modulations.
(PDF)

Table S4
**Fitting parameters corresponding to the SANS data recorded at the ILL (Fig. S1, curves** 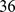 **–** 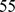 **), as obtained by both Guinier analysis and the QUAFIT method.** The related hemocyanin concentration is 10.0 gL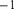 and pH is 7.0 for all the samples. Condition G: 50 mM phosphate, 10 mM sodium sulphite, 0.1 mM 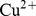; Condition H: 50 mM Tris/HCl, 10 mM sodium sulphite, 0.1 mM 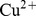; Condition I: 50 mM phosphate; Condition J: 50 mM Tris/HCl. The uncertainties on the 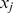 values affect the last reported decimal digit.(PDF)

Table S5
**Fitting parameters referring to the SAXS patterns recorded at the ESRF (Fig. S1, curves** 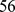 **–** 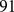 **), as obtained by both Guinier analysis and the QUAFIT method (pH 7.0 for all samples).** Condition K: hemocyanin concentration 1.0 gL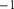, 50 mM Tris/HCl; Condition L: hemocyanin concentration 5.0 gL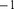, 50 mM Tris/HCl; Condition M: hemocyanin concentration 10.0 gL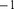, 50 mM Tris/HCl; Condition N: hemocyanin concentration 1.0 gL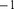, 50 mM Tris/HCl, 10 mM sodium sulphite; Condition O: hemocyanin concentration 5.0 gL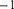, 50 mM Tris/HCl, 10 mM sodium sulphite; Condition P: hemocyanin concentration 10.0 gL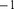, 50 mM Tris/HCl, 10 mM sodium sulphite, Condition Q: hemocyanin concentration 1.0 gL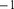, 50 mM phosphate; Condition R: hemocyanin concentration 5.0 gL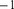, 50 mM phosphate; Condition S: hemocyanin concentration 10.0 gL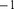, 50 mM phosphate; Condition T: hemocyanin concentration 1.0 gL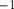, 50 mM phosphate, 10 mM sodium sulphite; Condition U: hemocyanin concentration 5.0 gL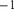, 50 mM phosphate, 10 mM sodium sulphite; Condition V: hemocyanin concentration 10.0 gL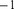, 50 mM phosphate, 10 mM sodium sulphite. The uncertainties on the 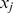 values affect the last reported decimal digit.(PDF)
