# Supplementary material for: Quaternary Structure Heterogeneity of Oligomeric Proteins: A SAXS and SANS Study of the Dissociation Products of Octopus vulgaris Hemocyanin
Source: PLoS One. 2012 Nov 15;7(11):e49644. doi: 10.1371/journal.pone.0049644 (PMC3499515; doi:10.1371/journal.pone.0049644)
Supplement: Table S1 — Detailed list of all chemical-physical conditions of the samples measured during the LURE and ESRF/1 campaigns. Red and blue colours highlight experimental conditions with the strongest and the lowest modulations, respectively [8]. (PDF) [file pone.0049644.s003.pdf]

#	Experiment	c gL^{-1}	pH	x_D	[Tris] mM	[P] mM	[Gly] mM	[Ca^{2+}] mM	[SO_3^{2-}] mM	[$S_2O_4^{2-}$] mM	[SO_4^{2-}] mM	[Cu^{2+}] mM	[EDTA] mM	[SCN^-] mM	[F^-] mM
1	LURE	10.0	7.0	0.00	50	0	0	40	5	5	0	0.1	0	0	0
2	LURE	10.0	7.0	0.00	50	0	0	40	0	0	10	0.1	0	0	0
3	ESRF/1	1.0	7.0	0.00	0	50	0	0	0	0	0	0	0	0	0
4	ESRF/1	1.0	7.0	0.00	50	0	0	0	0	0	0	0	0	0	0
5	ESRF/1	2.5	7.0	0.00	50	0	0	0	0	0	0	0	0	0	0
6	ESRF/1	5.0	7.0	0.00	50	0	0	0	0	0	0	0	0	0	0
7	ESRF/1	7.5	7.0	0.00	50	0	0	0	0	0	0	0	0	0	0
8	ESRF/1	10.0	7.0	0.00	50	0	0	0	0	0	0	0	0	0	0
9	ESRF/1	1.0	7.0	0.00	0	50	0	0	0	5	0	0	0	0	0
10	ESRF/1	2.5	7.0	0.00	0	50	0	0	0	5	0	0	0	0	0
11	ESRF/1	5.0	7.0	0.00	0	50	0	0	0	5	0	0	0	0	0
12	ESRF/1	7.5	7.0	0.00	0	50	0	0	0	5	0	0	0	0	0
13	ESRF/1	10.0	7.0	0.00	0	50	0	0	0	5	0	0	0	0	0
14	ESRF/1	1.0	7.0	0.00	50	0	0	0	0	5	0	0	0	0	0
15	ESRF/1	2.5	7.0	0.00	50	0	0	0	0	5	0	0	0	0	0
16	ESRF/1	5.0	7.0	0.00	50	0	0	0	0	5	0	0	0	0	0
17	ESRF/1	7.5	7.0	0.00	50	0	0	0	0	5	0	0	0	0	0
18	ESRF/1	10.0	7.0	0.00	50	0	0	0	0	5	0	0	0	0	0
19	ESRF/1	5.0	9.5	0.00	0	0	50	0	0	0	0	0	5	100	0
20	ESRF/1	5.0	9.5	0.00	0	0	50	0	0	0	0	0	5	100	50
21	ESRF/1	5.0	9.5	0.00	0	0	50	0	0	0	0	0	5	100	100
22	ESRF/1	5.0	9.5	0.00	0	0	50	0	0	0	0	0	5	100	150
23	ESRF/1	5.0	9.5	0.00	0	0	50	0	0	0	0	0	5	100	200
24	ESRF/1	5.0	9.5	0.00	0	0	50	0	0	0	0	0	5	100	250
25	ESRF/1	5.0	9.5	0.00	0	0	50	0	0	0	0	0	5	100	300
26	ESRF/1	5.0	9.5	0.00	0	0	50	0	0	0	0	0	5	100	390
27	ESRF/1	5.0	7.0	0.00	0	50	0	0	0	0	0	0	0	100	0
28	ESRF/1	5.0	7.0	0.00	0	50	0	0	0	0	0	0	0	100	50
29	ESRF/1	5.0	7.0	0.00	0	50	0	0	0	0	0	0	0	100	150
30	ESRF/1	5.0	7.0	0.00	0	50	0	0	0	0	0	0	0	100	250
31	ESRF/1	5.0	7.0	0.00	50	0	0	0	0	0	0	0	0	100	0
32	ESRF/1	5.0	7.0	0.00	50	0	0	0	0	0	0	0	0	100	50
33	ESRF/1	5.0	7.0	0.00	50	0	0	0	0	0	0	0	0	100	150
34	ESRF/1	5.0	7.0	0.00	50	0	0	0	0	0	0	0	0	100	250
35	ESRF/1	5.0	7.0	0.00	50	0	0	0	0	0	0	0	0	100	280

Table S1:
