# Supplementary material for: Quaternary Structure Heterogeneity of Oligomeric Proteins: A SAXS and SANS Study of the Dissociation Products of Octopus vulgaris Hemocyanin
Source: PLoS One. 2012 Nov 15;7(11):e49644. doi: 10.1371/journal.pone.0049644 (PMC3499515; doi:10.1371/journal.pone.0049644)
Supplement: Table S2 — Detailed list of all chemical-physical conditions of the samples measured at the ILL. (PDF) [file pone.0049644.s004.pdf]

#	Experiment	c gL^{-1}	pH	x_D	[Tris] mM	[P] mM	[Gly] mM	$[Ca^{2+}]$ mM	$[SO_3^{2-}]$ mM	$[S_2O_4^{2-}]$ mM	$[SO_4^{2-}]$ mM	$[Cu^{2+}]$ mM	[EDTA] mM	$[SCN^-]$ mM	$[F^-]$ mM
36	ILL	10.0	7.0	0.00	0	50	0	0	10	0	0	0.1	0	0	0
37	ILL	10.0	7.0	0.00	50	0	0	0	10	0	0	0.1	0	0	0
38	ILL	10.0	7.0	0.00	0	50	0	0	0	0	0	0	0	0	0
39	ILL	10.0	7.0	0.00	50	0	0	0	0	0	0	0	0	0	0
40	ILL	10.0	7.0	0.25	0	50	0	0	10	0	0	0.1	0	0	0
41	ILL	10.0	7.0	0.25	50	0	0	0	10	0	0	0.1	0	0	0
42	ILL	10.0	7.0	0.25	0	50	0	0	0	0	0	0	0	0	0
43	ILL	10.0	7.0	0.25	50	0	0	0	0	0	0	0	0	0	0
44	ILL	10.0	7.0	0.50	0	50	0	0	10	0	0	0.1	0	0	0
45	ILL	10.0	7.0	0.50	50	0	0	0	10	0	0	0.1	0	0	0
46	ILL	10.0	7.0	0.50	0	50	0	0	0	0	0	0	0	0	0
47	ILL	10.0	7.0	0.50	50	0	0	0	0	0	0	0	0	0	0
48	ILL	10.0	7.0	0.75	0	50	0	0	10	0	0	0.1	0	0	0
49	ILL	10.0	7.0	0.75	50	0	0	0	10	0	0	0.1	0	0	0
50	ILL	10.0	7.0	0.75	0	50	0	0	0	0	0	0	0	0	0
51	ILL	10.0	7.0	0.75	50	0	0	0	0	0	0	0	0	0	0
52	ILL	10.0	7.0	1.00	0	50	0	0	10	0	0	0.1	0	0	0
53	ILL	10.0	7.0	1.00	50	0	0	0	10	0	0	0.1	0	0	0
54	ILL	10.0	7.0	1.00	0	50	0	0	0	0	0	0	0	0	0
55	ILL	10.0	7.0	1.00	50	0	0	0	0	0	0	0	0	0	0

Table S2:
