# Supplementary material for: Quaternary Structure Heterogeneity of Oligomeric Proteins: A SAXS and SANS Study of the Dissociation Products of Octopus vulgaris Hemocyanin
Source: PLoS One. 2012 Nov 15;7(11):e49644. doi: 10.1371/journal.pone.0049644 (PMC3499515; doi:10.1371/journal.pone.0049644)
Supplement: Table S3 — Detailed list of all chemical-physical conditions of the samples measured during the ESRF/2 campaign. Red colours highlight experimental conditions with the strongest modulations. (PDF) [file pone.0049644.s005.pdf]

#	Experiment	c gL^{-1}	pH	x_D	[Tris] mM	[P] mM	[Gly] mM	[Ca^{2+}] mM	[SO_3^{2-}] mM	[$S_2O_4^{2-}$] mM	[SO_4^{2-}] mM	[Cu^{2+}] mM	[EDTA] mM	[SCN^-] mM	[F^-] mM
56	ESRF/2	1.0	7.0	0.00	50	0	0	0	0	0	0	0	0	0	0
57	ESRF/2	1.0	7.0	0.50	50	0	0	0	0	0	0	0	0	0	0
58	ESRF/2	1.0	7.0	1.00	50	0	0	0	0	0	0	0	0	0	0
59	ESRF/2	5.0	7.0	0.00	50	0	0	0	0	0	0	0	0	0	0
60	ESRF/2	5.0	7.0	0.50	50	0	0	0	0	0	0	0	0	0	0
61	ESRF/2	5.0	7.0	1.00	50	0	0	0	0	0	0	0	0	0	0
62	ESRF/2	10.0	7.0	0.00	50	0	0	0	0	0	0	0	0	0	0
63	ESRF/2	10.0	7.0	0.50	50	0	0	0	0	0	0	0	0	0	0
64	ESRF/2	10.0	7.0	1.00	50	0	0	0	0	0	0	0	0	0	0
65	ESRF/2	1.0	7.0	0.00	50	0	0	0	10	0	0	0	0	0	0
66	ESRF/2	1.0	7.0	0.50	50	0	0	0	10	0	0	0	0	0	0
67	ESRF/2	1.0	7.0	1.00	50	0	0	0	10	0	0	0	0	0	0
68	ESRF/2	5.0	7.0	0.00	50	0	0	0	10	0	0	0	0	0	0
69	ESRF/2	5.0	7.0	0.50	50	0	0	0	10	0	0	0	0	0	0
70	ESRF/2	5.0	7.0	1.00	50	0	0	0	10	0	0	0	0	0	0
71	ESRF/2	10.0	7.0	0.00	50	0	0	0	10	0	0	0	0	0	0
72	ESRF/2	10.0	7.0	0.50	50	0	0	0	10	0	0	0	0	0	0
73	ESRF/2	10.0	7.0	1.00	50	0	0	0	10	0	0	0	0	0	0
74	ESRF/2	1.0	7.0	0.00	0	50	0	0	0	0	0	0	0	0	0
75	ESRF/2	1.0	7.0	0.50	0	50	0	0	0	0	0	0	0	0	0
76	ESRF/2	1.0	7.0	1.00	0	50	0	0	0	0	0	0	0	0	0
77	ESRF/2	5.0	7.0	0.00	0	50	0	0	0	0	0	0	0	0	0
78	ESRF/2	5.0	7.0	0.50	0	50	0	0	0	0	0	0	0	0	0
79	ESRF/2	5.0	7.0	1.00	0	50	0	0	0	0	0	0	0	0	0
80	ESRF/2	10.0	7.0	0.00	0	50	0	0	0	0	0	0	0	0	0
81	ESRF/2	10.0	7.0	0.50	0	50	0	0	0	0	0	0	0	0	0
82	ESRF/2	10.0	7.0	1.00	0	50	0	0	0	0	0	0	0	0	0
83	ESRF/2	1.0	7.0	0.00	0	50	0	0	10	0	0	0	0	0	0
84	ESRF/2	1.0	7.0	0.50	0	50	0	0	10	0	0	0	0	0	0
85	ESRF/2	1.0	7.0	1.00	0	50	0	0	10	0	0	0	0	0	0
86	ESRF/2	5.0	7.0	0.00	0	50	0	0	10	0	0	0	0	0	0
87	ESRF/2	5.0	7.0	0.50	0	50	0	0	10	0	0	0	0	0	0
88	ESRF/2	5.0	7.0	1.00	0	50	0	0	10	0	0	0	0	0	0
89	ESRF/2	10.0	7.0	0.00	0	50	0	0	10	0	0	0	0	0	0
90	ESRF/2	10.0	7.0	0.50	0	50	0	0	10	0	0	0	0	0	0
91	ESRF/2	10.0	7.0	1.00	0	50	0	0	10	0	0	0	0	0	0

Table S3:
