# Supplementary material for: Quaternary Structure Heterogeneity of Oligomeric Proteins: A SAXS and SANS Study of the Dissociation Products of Octopus vulgaris Hemocyanin
Source: PLoS One. 2012 Nov 15;7(11):e49644. doi: 10.1371/journal.pone.0049644 (PMC3499515; doi:10.1371/journal.pone.0049644)
Supplement: Table S4 — Fitting parameters corresponding to the SANS data recorded at the ILL (Fig. S1, curves – ), as obtained by both Guinier analysis and the QUAFIT method. The related hemocyanin concentration is 10.0 gL and pH is 7.0 for all the samples. Condition G: 50 mM phosphate, 10 mM sodium sulphite, 0.1 mM ; Condition H: 50 mM Tris/HCl, 10 mM sodium sulphite, 0.1 mM ; Condition I: 50 mM phosphate; Condition J: 50 mM Tris/HCl. The uncertainties on the values affect the last reported decimal digit. (PDF) [file pone.0049644.s006.pdf]

	R_g (Å)	$\frac{d\Sigma}{d\Omega}(0)$ (cm^{-1})	$<N_{agg}>_G$	$<N_{agg}>_Q$	χ^2	B (10^{-3} cm^{-1})	x_{10}	x_8	x_6	x_4	x_2	x_1	x_{1_L}	Fig.	Experiment
Condition G															
x_D=0.00	134±3	10.5±0.3	5.6±0.2	5.5±0.4	14.1	0	0.08	0.39	0.21	0	0	0	0.32	S1-36	36-ILL
x_D=0.25	134±4	1.47±0.06	4.0±0.3	3±1	16.7	0	0.2	0	0	0	0	0	0.8	S1-40	40-ILL
x_D=0.50	132±9	0.15±0.01	7±4	7±3	4.9	0	0.4	0.1	0.1	0.4	0	0	0	S1-44	44-ILL
x_D=0.75	134±3	6.0±0.2	7±1	7.4±0.6	13.7	5.0±0.5	0.56	0	0	0.44	0	0	0	S1-48	48-ILL
x_D=1.00	138±3	18.1±0.6	6.6±0.7	6.2±0.2	16.3	0	0.34	0	0.43	0	0	0	0.23	S1-52	52-ILL
Condition H															
x_D=0.00	124±2	6.8±0.1	3.63±0.08	3.9±0.4	15.1	0	0	0.29	0.1	0.12	0	0	0.48	S1-37	37-ILL
x_D=0.25	126±3	1.24±0.03	3.4±0.2	3±1	4.2	0	0.06	0.1	0.2	0.1	0	0	0.6	S1-41	41-ILL
x_D=0.50	180±30	0.3±0.1	13±8	7±3	7.2	0.71±0.07	0.5	0	0	0.5	0	0	0	S1-45	45-ILL
x_D=0.75	91±1	1.94±0.03	2.4±0.3	2.6±0.5	9.3	1.4±0.2	0	0	0	0.55	0	0.5	0	S1-49	49-ILL
x_D=1.00	117±2	11.7±0.2	4.2±0.4	3.9±0.2	17.7	14±1	0.13	0	0	0.6	0	0.28	0	S1-53	53-ILL
Condition I															
x_D=0.00	102±1	4.71±0.06	2.53±0.04	2.5±0.3	3.7	1.4±0.1	0	0.01	0.02	0.33	0.4	0.01	0.25	S1-38	38-ILL
x_D=0.25	125±2	2.20±0.05	6.0±0.3	6±2	15.0	0	0.2	0	0	0.7	0	0	0	S1-42	42-ILL
x_D=0.50	132±10	0.15±0.01	7±4	6±2	15.3	0	0.2	0	0.4	0.4	0	0	0	S1-46	46-ILL
x_D=0.75	119±2	4.21±0.07	5.2±0.7	5.9±0.5	8.3	5.2±0.5	0.38	0	0	0.52	0	0.1	0	S1-50	50-ILL
x_D=1.00	128±3	16.9±0.5	6.1±0.7	6.0±0.2	12.5	17±2	0.37	0	0	0.56	0	0.07	0	S1-54	54-ILL
Condition J															
x_D=0.00	75±1	2.57±0.03	1.38±0.02	1.7±0.4	16.2	0	0	0	0	0	0.7	0.03	0.3	S1-39	39-ILL
x_D=0.25	85±2	0.74±0.01	2.0±0.1	2±1	9.8	0	0	0	0	0.1	0.6	0.3	0	S1-43	43-ILL
x_D=0.50	89±9	0.051±0.005	2±1	3±3	2.5	0	0	0	0	0.6	0	0.4	0	S1-47	47-ILL
x_D=0.75	89±1	2.09±0.02	2.6±0.4	3.2±0.4	13.1	6.7±0.7	0	0	0	0.7	0	0.28	0	S1-51	51-ILL
x_D=1.00	91±1	7.52±0.08	2.7±0.3	3.16±0.07	13.0	18±2	0	0	0	0.72	0	0.28	0	S1-55	55-ILL

Table S4:
