# Supplementary material for: Quaternary Structure Heterogeneity of Oligomeric Proteins: A SAXS and SANS Study of the Dissociation Products of Octopus vulgaris Hemocyanin
Source: PLoS One. 2012 Nov 15;7(11):e49644. doi: 10.1371/journal.pone.0049644 (PMC3499515; doi:10.1371/journal.pone.0049644)
Supplement: Table S5 — Fitting parameters referring to the SAXS patterns recorded at the ESRF (Fig. S1, curves – ), as obtained by both Guinier analysis and the QUAFIT method (pH 7.0 for all samples). Condition K: hemocyanin concentration 1.0 gL, 50 mM Tris/HCl; Condition L: hemocyanin concentration 5.0 gL, 50 mM Tris/HCl; Condition M: hemocyanin concentration 10.0 gL, 50 mM Tris/HCl; Condition N: hemocyanin concentration 1.0 gL, 50 mM Tris/HCl, 10 mM sodium sulphite; Condition O: hemocyanin concentration 5.0 gL, 50 mM Tris/HCl, 10 mM sodium sulphite; Condition P: hemocyanin concentration 10.0 gL, 50 mM Tris/HCl, 10 mM sodium sulphite, Condition Q: hemocyanin concentration 1.0 gL, 50 mM phosphate; Condition R: hemocyanin concentration 5.0 gL, 50 mM phosphate; Condition S: hemocyanin concentration 10.0 gL, 50 mM phosphate; Condition T: hemocyanin concentration 1.0 gL, 50 mM phosphate, 10 mM sodium sulphite; Condition U: hemocyanin concentration 5.0 gL, 50 mM phosphate, 10 mM sodium sulphite; Condition V: hemocyanin concentration 10.0 gL, 50 mM phosphate, 10 mM sodium sulphite. The uncertainties on the values affect the last reported decimal digit. (PDF) [file pone.0049644.s007.pdf]

	R_g (Å)	$\frac{d\Sigma}{d\Omega}(0)$ (cm^{-1})	$<N_{agg}>_G$	$<N_{agg}>_Q$	χ^2	B (10^{-3} cm^{-1})	x_{10}	x_8	x_6	x_4	x_2	x_1	x_{1_L}	Fig.	Experiment
				Condition K											
x_D=0.00	60.1±0.5	0.323±0.003	1.2±0.2	1.3±0.2	0.1	0.26±0.03	0	0.04	0	0	0	0.7	0.3	S1-56	56-ESRF/2
x_D=0.50	85±1	0.565±0.008	2.1±0.3	2.2±0.3	1.3	1.6±0.1	0.02	0.04	0.09	0.08	0	0.77	0	S1-57	57-ESRF/2
x_D=1.00	102.5±0.7	0.895±0.007	3.3±0.5	3.3±0.3	4.1	2.6±0.3	0.14	0	0	0.35	0	0.51	0	S1-58	58-ESRF/2
				Condition L											
x_D=0.00	68.8±0.7	2.22±0.02	1.6±0.3	1.75±0.09	8.2	8.4±0.8	0.01	0.01	0.04	0.13	0	0.81	0	S1-59	59-ESRF/2
x_D=0.50	90.5±0.9	3.51±0.03	2.6±0.4	2.7±0.1	13.3	11±1	0.06	0.01	0	0.33	0	0.59	0	S1-60	60-ESRF/2
x_D=1.00	105.2±0.8	4.28±0.03	3.2±0.5	3.0±0.3	2.9	25±2	0	0.2	0.01	0.03	0.47	0	0.29	S1-61	61-ESRF/2
				Condition M											
x_D=0.00	72.2±0.6	4.79±0.04	1.8±0.3	1.80±0.06	14.6	18±2	0	0	0.02	0.22	0	0.76	0	S1-62	62-ESRF/2
x_D=0.50	90.2±0.7	7.52±0.06	2.8±0.4	2.73±0.08	15.2	29±3	0.02	0	0	0.5	0	0.47	0	S1-63	63-ESRF/2
x_D=1.00	101.6±0.6	9.22±0.06	3.4±0.5	3.3±0.1	17.7	26±3	0.09	0	0	0.48	0	0.42	0	S1-64	64-ESRF/2
				Condition N											
x_D=0.00	134.0±0.4	2.039±0.009	8±1	6.8±0.5	0.7	2.7±0.3	0.62	0	0	0.05	0.03	0.28	0.01	S1-65	65-ESRF/2
x_D=0.50	132.1±0.5	1.855±0.009	7±1	6.2±0.5	0.6	2.7±0.2	0.56	0	0	0.02	0.17	0.22	0.03	S1-66	66-ESRF/2
x_D=1.00	139.4±0.7	2.82±0.02	10±2	9.3±0.8	2.0	3.8±0.4	0.9	0	0.1	0	0	0	0.03	S1-67	67-ESRF/2
				Condition O											
x_D=0.00	134.3±0.7	11.44±0.08	8±1	7.6±0.3	3.7	14±1	0.7	0	0	0.05	0.13	0.11	0	S1-68	68-ESRF/2
x_D=0.50	120.4±0.2	6.68±0.02	5.0±0.8	4.6±0.2	4.1	12±1	0.22	0.1	0	0.32	0	0.36	0	S1-69	69-ESRF/2
x_D=1.00	138.3±0.6	14.05±0.09	10±2	9.1±0.4	14.8	18±2	0.8	0	0.22	0	0	0	0	S1-70	70-ESRF/2
				Condition P											
x_D=0.00	130.0±0.8	19.6±0.2	7±1	6.4±0.1	14.4	22±2	0.54	0	0	0.16	0.07	0.12	0.1	S1-71	71-ESRF/2
x_D=0.50	132.3±0.8	24.8±0.2	9±1	8.1±0.1	18.4	37±3	0.69	0	0	0.31	0	0	0	S1-72	72-ESRF/2
x_D=1.00	133.6±0.6	24.3±0.2	9±1	7.9±0.1	16.6	34±4	0.59	0	0.2	0.21	0	0	0	S1-73	73-ESRF/2
				Condition Q											
x_D=0.00	104.5±0.8	0.691±0.006	2.5±0.4	2.3±0.2	0.1	1.00±0.09	0.1	0.02	0.02	0.06	0.1	0.37	0.36	S1-74	74-ESRF/2
x_D=0.50	112.1±0.4	0.936±0.004	3.5±0.5	3.2±0.3	0.4	2.1±0.2	0.1	0	0.23	0.07	0	0.48	0.13	S1-75	75-ESRF/2
x_D=1.00	122.3±0.6	1.484±0.009	5.5±0.8	5.2±0.3	3.7	3.7±0.4	0.31	0	0	0.5	0	0.23	0	S1-76	76-ESRF/2
				Condition R											
x_D=0.00	129.0±0.4	7.40±0.03	5.5±0.8	5.0±0.1	0.6	6.8±0.6	0.23	0.23	0.03	0.04	0.03	0.12	0.32	S1-77	77-ESRF/2
x_D=0.50	128.6±0.4	7.73±0.03	5.7±0.9	5.3±0.1	4.2	11±1	0.19	0.23	0.15	0.01	0.18	0.11	0.13	S1-78	78-ESRF/2
x_D=1.00	141±1	9.7±0.1	7±1	6.9±0.3	18.9	17±2	0	0.83	0	0.03	0	0.05	0.09	S1-79	79-ESRF/2
				Condition S											
x_D=0.00	128.2±0.4	14.04±0.06	5.2±0.8	4.5±0.4	0.5	3.9±0.4	0.37	0	0	0	0.15	0.15	0.31	S1-80	80-ESRF/2
x_D=0.50	124.1±0.4	15.48±0.06	5.7±0.9	5.1±0.2	6.1	22±3	0.35	0	0	0.33	0.02	0.23	0.08	S1-81	81-ESRF/2
x_D=1.00	117.9±0.3	13.30±0.03	4.9±0.8	4.52±0.08	8.3	24±2	0.16	0	0.21	0.32	0.08	0.16	0.08	S1-82	82-ESRF/2
				Condition T											
x_D=0.00	137.9±0.7	2.17±0.01	8±1	7.1±0.5	0.9	0.58±0.05	0.67	0	0	0	0	0	0.33	S1-83	83-ESRF/2
x_D=0.50	139.2±0.7	2.41±0.02	9±1	7.9±0.5	0.7	2.1±0.2	0.76	0	0	0	0	0	0.24	S1-84	84-ESRF/2
x_D=1.00	138.6±0.7	2.65±0.02	10±2	8.7±0.5	0.6	3.0±0.3	0.8	0	0.02	0.01	0.07	0	0.06	S1-85	85-ESRF/2
				Condition U											
x_D=0.00	134.9±0.8	11.05±0.08	8±1	6.7±0.2	6.0	8.0±0.8	0.63	0	0	0	0	0	0.37	S1-86	86-ESRF/2
x_D=0.50	136.9±0.8	12.06±0.09	9±1	8.0±0.2	3.2	9.2±0.9	0.77	0	0	0	0	0	0.23	S1-87	87-ESRF/2
x_D=1.00	134.3±0.5	10.95±0.06	8±1	7.3±0.3	1.6	12±1	0.64	0	0	0.17	0.01	0.05	0.12	S1-88	88-ESRF/2
				Condition V											
x_D=0.00	137.5±0.7	24.4±0.2	9±1	7.9±0.6	1.8	6.3±0.7	0.7	0	0	0.04	0	0	0.21	S1-89	89-ESRF/2
x_D=0.50	134.1±0.9	24.9±0.2	9±1	8.1±0.2	16.7	25±3	0.75	0	0	0.13	0.03	0	0.09	S1-90	90-ESRF/2
x_D=1.00	134.1±0.8	23.8±0.2	9±1	7.8±0.2	6.7	24±3	0.69	0	0	0.19	0	0	0.12	S1-91	91-ESRF/2

Table S5:
